# COVID-19 pandemic repercussions on undergraduate teaching in obstetrics and gynecology

**DOI:** 10.1016/j.clinsp.2022.100025

**Published:** 2022-03-07

**Authors:** Lívia Sousa Ribeiro, Rossana Pulcineli Vieira Francisco, Fábio Roberto Cabar

**Affiliations:** a Centro de Ciências da Saúde, Escola de Medicina ‒ Universidade Federal do Recôncavo da Bahia, Cruz das Almas, BA, Brazil; b Faculdade de Medicina da Universidade de São Paulo, Department of Obstetrics and Gynecology, São Paulo, SP, Brazil

The COVID-19 coronavirus pandemic represents one of the greatest challenges in recent decades. In March 2020, the World Health Organization declared a Public Health Emergency of International Concern, declaring COVID-19 a pandemic.^1^ This serious crisis directly or indirectly affected various sectors of society, including the educational sector, whose operation was interrupted indefinitely. In this context, universities, colleges, and public and private schools were forced to suspend their activities.^2^^,^^3^ According to the United Nations Educational, Scientific, and Cultural Organization (UNESCO), this situation affected 90% of students worldwide.^4^ Debates arose in the academic environment about the implementation of alternatives that would allow the continuation of educational activities, especially those related to the training of health professionals while complying with the social isolation measures in force and the quality of teaching-learning.

## Insertion of remote education models

From this perspective, the initiatives included the development of new distance education platforms with the possibility of synchronous and asynchronous remote teaching, in addition to the use of question databases and other simulation resources.^5^ As a result, a significant difference was observed between the time spent on online platforms before and during COVID-19, with 7.4% of students using them before and 23.6% of students spending more than 15 hours a week during the pandemic.^6^ This change leads to the need to discuss this new teaching context and the benefits and harms to the learning of medical students.

Online teaching allowed medical training to continue in an atypical situation and its greatest benefit was the flexibility offered by the teaching platforms.^6^ A survey conducted in Brazil revealed that 80.8% of students considered it feasible to implement remote teaching for the continuation of their medical course, while 19.2% mentioned that it was not.^7^ In Jordan, the Middle East, the overall satisfaction rate for remote medical education was 26.8%, and it was significantly higher in students with previous experience in distance learning and when instructors actively participated in classes using devices such as multimedia and dedicating sufficient time to teaching.^8^

## Medical students learning impacts

Commonly perceived barriers to using online learning platforms included distraction caused by family members (26.8%) and a poor Internet connection (21.5%).^6^ Moreover, in a survey of 1,019 respondents, 32% reported being more stressed due to tests and exams performed online. The duration of the exam, how the questions were formulated, technical e-learning platform problems and internet connectivity were the main factors associated with stress in 78.1%, 76.3%, 63.5%, and 66.1% of these students, respectively. Other factors mentioned included the concern with the teaching methods used, the means of assessment, and the dishonesty of students during these exams.^9^

Another relevant issue is that although most pre-clinical experiences can be replaced by pre-recorded lectures and videoconference sessions, this is not completely true for practical, clinical, or surgical activities. These hands-on activities enable students to understand how multidisciplinary teams work and to practice newly learned clinical skills while understanding how the health system works.^10^ This practical experience can also influence the decision of their future specialty, in addition to significantly contributing to learning surgical disciplines, such as Gynecology and Obstetrics.

## Impairments in the teaching of Gynecology and Obstetrics

The National Commission Specialized in Medical Residency of the Brazilian Federation of Gynecology and Obstetrics Associations (*Federação Brasileira das Associações de Ginecologia e Obstetrícia* ‒ FEBRASGO) published a series of recommendations for standardizing teaching, reducing pedagogical losses, and ensuring the safety of medical residents. Regarding theoretical activities, the recommendation was to allocate the maximum workload (total of 12 hours per week) for theoretical activities, replacing face-to-face courses with technology-mediated distance learning as much as possible.^11^ Issues related to professional safety and care for patients with COVID-19 were included in the theoretical content. The recommendation for practical activities was to set the maximum workload at 48 hours per week, including different shifts. It is also recommended that, given the non-elective characteristic of obstetrics, activities such as prenatal care, childbirth, postpartum, and obstetric pathology wards continue. In Gynecology, essential activities such as cancer treatments, care for victims of sexual violence, and others such as care for sexually transmitted infections and family planning should continue according to the possibilities of each program.^12^

Despite this recommendation, a study found that graduate residents in Gynecology and Obstetrics were reallocated to activities not related to the specialty. They screened patients suspected of COVID-19 or provided care in COVID-19 wards.^13^ A total of 20% of the institutions relocated their Gynecology and Obstetrics residents to assist in ICUs for patients with COVID-19. The activities most affected included gynecological surgeries, of which 72% were canceled, followed by gynecological outpatient appointments, with 23% cancellations; 90% of elective surgeries were canceled in more than 50% of services.^13^ In addition to the cancellation of elective activities for undergraduate students, medical residents that presented a risk factor, such as being pregnant and/or contaminated, needed to distance themselves from practical activities, which made them feel uncertain and insecure about the possibility of filling this gap in their medical training in the future.^14^ This situation demonstrates that the teaching of Gynecology and Obstetrics may have been impaired, directly affecting medical students who were interested in this specialty and wanted to have a practical experience on it during the course.

## The concern of medical students regarding specialties of interest

This was found to be especially true for primarily surgical disciplines. In a survey with 238 medical students interested in Urology, mainly attending the third and fourth years, 82% reported a decrease in exposure to practical activities focused on the specialty. Nearly half reported changes to required rotations, and 35% reported changes to specific Urology rotations at their home institutions. Nevertheless, 85% reported that the pandemic did not prevent them from choosing their specialty.^15^ Similarly, another survey conducted with students interested in Neurosurgery found that 63% were concerned about conferences and networking opportunities, 59% about clinical experiences, and 42% about board exam scores. Among third-year students, 76% reported that one or more neurosurgery rotations were canceled or postponed during the pandemic.^16^ Another survey conducted with medical students interested in Plastic Surgery found that they perceived a negative impact of the COVID-19 pandemic on their education due to a reduction in exposure to clinical experience.^17^

The results of these studies highlight the concerns of medical students on medical learning and training, especially regarding their specialties of interest, during the COVID-19 pandemic. It should be noted that, until now, there is not a single Brazilian study assessing the impact of the pandemic on the training in Obstetrics and Gynecology of undergraduate medical students. Although Brito et al. (2020) have highlighted the potential impact of the COVID-19 pandemic for this specialty, this study focused on the impacts on the training of medical residents.

Future studies should fill this knowledge gap, thus contributing to the development of strategies to address the deficiencies generated by the pandemic, given the relevance and need for an undergraduate curriculum that includes practical knowledge in Obstetrics and Gynecology.^18^ It is a basic and essential specialty in medical education, which directly impacts the quality of health care in Brazil in the future.

The teaching of Obstetrics and Gynecology depends on surgical and practical activities; the changes in educational strategies in the teaching of this field of medical knowledge caused by the COVID-19 pandemic may have compromised the undergraduate students’ learnings.

## Authors’ contributions

The authors contributed equally to the study. They were responsible for the conception and design, manuscript writing and revision, approval of the final version of the manuscript.

## Conflicts of interest

The authors declare no conflicts of interest.

## References

[1] Coronavirus disease (COVID-19) – World Health Organization [Internet]. [cited 2021 Nov 10]. Available from: https://www.who.int/emergencies/diseases/novel-coronavirus-2019.

[2] Vercelli L de CA (2020). Aulas remotas em tempos de COVID-19: a percepção de discentes de um programa de mestrado profissional em educação. Rev Mbienteeducação.

[3] Bezerra ACV, da Silva CEM, Soares FRG, da Silva JAM. (2020). Fatores associados ao comportamento da população durante o isolamento social na pandemia de COVID-19. Ciênc Saúde Colet.

[4] Organização das Nações Unidas para a Educação, a Ciência e a Cultura. A Comissão Futuros da Educação da Unesco apela ao planejamento antecipado contra o aumento das desigualdades após a COVID-19. Unesco: 2020 [cited 2021 Nov 10]. Available from: https://pt.unesco.org/news/comissao-futuros-da-educacao-da-unesco-apela-ao-planejamentoantecipado-o-aumento-das.

[5] Alves L (2020). Educação remota: entre a ilusão e a realidade. Edu.

[6] Dost S, Hossain A, Shehab M, Abdelwahed A, Al-Nusair L. (2020). Perceptions of medical students towards online teaching during the COVID-19 pandemic: a national cross-sectional survey of 2721 UK medical students. BMJ Open.

[7] Silva PHS, Faustino LR, Oliveira Sobrinho MS, Silva FBF (2021). Educação remota na continuidade da formação médica em tempos de pandemia: viabilidade e percepções. Rev Bras Educ Med.

[8] Al-Balas M, HI Al-Balas, Jaber HM, Obeidat K, Al-Balas H, Aborajooh EA (2020). Distance learning in clinical medical education amid COVID-19 pandemic in Jordan: current situation, challenges, and perspectives. BMC Med Educ.

[9] Elsalem L, Al-Azzam N, Jum'ah AA, Obeidat N, Sindiani AM, Kheirallah KA (2020). Stress and behavioral changes with remote E-exams during the COVID-19 pandemic: A cross-sectional study among undergraduates of medical sciences. Ann Med Surg.

[10] Hilburg R, Patel N, Ambruso S, Biewald MA, Farouk SS. (2020). Medical education during the coronavirus disease-2019 pandemic: learning from a distance. Adv Chronic Kidney Dis.

[11] Ministério da Educação. Nota Técnica no. 1/2020/CNRM/CGRS/DDES/SESU/SESU. Recomendações quanto ao desenvolvimento das atividades dos Programas de Residência Médica (PRMs) durante enfrentamento à pandemia por COVID-19. 2020 [cited 2021 Nov 10]. Available from: http://portal.mec.gov.br/index.php?option=com_docman&view=download&alias=145481-sei-23000&category_slug=2020&Itemid=30192» http://portal.mec.gov.br/index.php?option=com_docman&view=download&alias=145481-sei-23000&category_slug=2020&Itemid=30192.

[12] Romão GS, Schreiner L, Laranjeiras CLS, Bella ZIKJD, Coelho RA, Simões MDCR (2020). Medical Residency in Gynecology and Obstetrics in Times of COVID-19: Recommendations of the National Specialized Commission on Medical Residency of FEBRASGO. Rev Bras Ginecol Obstet.

[13] Brito LGO, Romão GS, Fernandes CE, Silva-Filho AL. (2020). Impact of COVID-19 on Brazilian medical residencies in obstetrics and gynecology. Int J Gynaecol Obstet Off Organ Int Fed Gynaecol Obstet.

[14] Simões MCR, Primo WQSP, Jakobi HR, Duran TCAL, Heinen BG. (2021). Rodízio dos médicos-residentes em ginecologia e obstetrícia durante a pandemia de COVID-19. Femina.

[15] Hanson KA, Borofsky MS, Hampson LA, Breyer BN, Kern NG, Conti SL (2020). Capturing the perspective of prospective urology applicants: impacts of COVID-19 on medical education. Urology.

[16] Guadix SW, Winston GM, Chae JK, Haghdel A, Chen J, Younus I (2020). Medical student concerns relating to neurosurgery education during COVID-19. World Neurosurg.

[17] Haley C, Lee J, Xun H, Yesantharao P, Nolan IT, Harirah M (2021). The negative impact of COVID-19 on medical education among medical students interested in plastic surgery: a cross-sectional survey study. Plast Reconstr Surg Glob Open.

[18] Salman H. (2021). Most significant barriers and proposed solutions for medical schools to facilitate simulation-based undergraduate curriculum in OBGYN. Arch Gynecol Obstet.

